# Electrophysiological Analysis of Brain Organoids: Current Approaches and Advancements

**DOI:** 10.3389/fnins.2020.622137

**Published:** 2021-01-12

**Authors:** Austin P. Passaro, Steven L. Stice

**Affiliations:** ^1^Regenerative Bioscience Center, University of Georgia, Athens, GA, United States; ^2^Division of Neuroscience, Biomedical & Health Sciences Institute, University of Georgia, Athens, GA, United States; ^3^Department of Animal and Dairy Science, University of Georgia, Athens, GA, United States

**Keywords:** electrophysiology, multi-electrode array, brain organoids, optogenetics, electrophysiological analysis, neurological disease modeling

## Abstract

Brain organoids, or cerebral organoids, have become widely used to study the human brain *in vitro*. As pluripotent stem cell-derived structures capable of self-organization and recapitulation of physiological cell types and architecture, brain organoids bridge the gap between relatively simple two-dimensional human cell cultures and non-human animal models. This allows for high complexity and physiological relevance in a controlled *in vitro* setting, opening the door for a variety of applications including development and disease modeling and high-throughput screening. While technologies such as single cell sequencing have led to significant advances in brain organoid characterization and understanding, improved functional analysis (especially electrophysiology) is needed to realize the full potential of brain organoids. In this review, we highlight key technologies for brain organoid development and characterization, then discuss current electrophysiological methods for brain organoid analysis. While electrophysiological approaches have improved rapidly for two-dimensional cultures, only in the past several years have advances been made to overcome limitations posed by the three-dimensionality of brain organoids. Here, we review major advances in electrophysiological technologies and analytical methods with a focus on advances with applicability for brain organoid analysis.

## Introduction

Over the past few decades, advances in stem cell biology have provided significant insight into neural development and understanding neurological disorders (Kelava and Lancaster, 2016b). Human pluripotent stem cells (hPSCs), especially human induced PSCs (hiPSCs), have proven very useful for modeling neurological disorders *in vitro* and examining potential therapeutics (Ebert et al., 2012; Avior et al., 2016; Liu et al., 2018). Recently, these models have improved with the advent of brain, or cerebral, organoids—three-dimensional self-organized structures containing many cell types and cytoarchitectures typical of the human brain (Lancaster et al., 2013; Kelava and Lancaster, 2016a).

Brain organoids have advanced quickly in complexity, from relatively unpredictable heterogeneous spheroids to highly organized and controllable representations of specific brain regions. This increased complexity can be attributed largely to advances and the coalescence of various technologies from many fields, such as biomaterials and genetics. To develop organoids in three dimensions, extracellular matrices—both organic and synthetic—and other scaffolds are vital to ensuring organoids have support to organize properly (Yin et al., 2016). Additionally, these materials play important roles in cell signaling and providing appropriate biomechanical cues needed for development (Yin et al., 2016).

Improved genetic technologies and transcriptomics, such as single-cell RNA sequencing, have allowed for detailed characterization of cell types and developmental states within organoids (Quadrato et al., 2017; Fischer et al., 2019); however, functional analysis of brain organoids is limited (Schröter et al., 2018; Poli et al., 2019). Classical electrophysiological methods such as patch clamp allow for high temporal resolution of neural activity in organoids but offer little spatial resolution for assessment of whole-organoid activity (Pasca et al., 2015). Calcium imaging provides larger-scale activity information but sacrifices temporal resolution and is reliant on imaging capabilities (Lancaster et al., 2013). Finally, microelectrode arrays (MEAs), adopted over the past several years (Giandomenico et al., 2019), provide both network-scale and high temporal resolution but currently lack three-dimensionality needed to properly analyze brain organoid activity. These and other key technologies for brain organoids are highlighted in this review with the goal of discussing how brain organoids have evolved so quickly in recent years, where the field has been slower to evolve, and looking forward to cutting-edge technologies with potential to overcome these shortcomings, primarily in electrophysiology. Here we directly compare strengths and weaknesses of existing and new electrophysiological methods, as they relate to organoid analysis.

## Brain Organoid Applications

Early brain organoids consisted of relatively disorganized, spontaneously differentiated structures containing multiple cell types characteristic of the human brain (Lancaster et al., 2013; Lancaster and Knoblich, 2014). While these primitive organoids proved useful for studying early aspects of development, such as neural migration, a lack of controlled differentiation and organization hindered reproducibility and more complex applications (Kelava and Lancaster, 2016b; Kyrousi and Cappello, 2020). Since then, more mature and organized brain organoids have been developed, allowing for a wide variety of developmental studies (Qian et al., 2019). While early brain organoids contributed primarily to developmental studies regarding neural stem maintenance and differentiation and corticogenesis (Kadoshima et al., 2013; Lancaster et al., 2013; Lancaster and Knoblich, 2014; Camp et al., 2015), later brain region-specific organoids provided insight into specific regional development, including both general regions (i.e., forebrain, midbrain) and highly specific regions and structures (i.e., hippocampus, cerebellum, retina) (Muguruma et al., 2015; Sakaguchi et al., 2015; Jo et al., 2016; Parfitt et al., 2016; Monzel et al., 2017; Muguruma, 2017). For a more comprehensive review of regional brain organoids, see Gopalakrishnan (2019).

The advent of brain region-specific organoids unlocked the potential for significantly improved neurological disease modeling (Jo et al., 2016; Monzel et al., 2017). The promise of stem cells, especially hiPSCs, for modeling neurodegenerative diseases has been acknowledged for years but somewhat hindered by traditional two-dimensional cell culture and difficult co-culture conditions. Two-dimensional cell culture does not allow for complex cellular interactions that occur in three dimensions *in vivo* and does not allow for analysis of certain disease phenotypes, such as extracellular protein aggregation in Alzheimer’s disease (Raja et al., 2016). By allowing for physiologically accurate, three-dimensional recapitulation of specific brain regions, more relevant models can be developed to study and develop therapeutics for diseases such as Parkinson’s disease and Alzheimer’s using brain organoids (Raja et al., 2016; Smits et al., 2019). For example, brain organoids generated from hiPSCs from Parkinson’s (Smits et al., 2019) and Alzheimer’s (Raja et al., 2016) patients recapitulate hallmark disease phenotypes, most notably reduced dopaminergic neurons in Parkinson’s organoids and amyloid beta aggregation and hyperphosphorylated tau protein in Alzheimer’s organoids. Similarly, as organoids are developed from hiPSCs, they may be used for personalized medicine to develop custom therapies for the above-mentioned diseases and other disorders (Kyrousi and Cappello, 2020). As evidence of this potential, organoids developed from several Alzheimer’s patients carrying different mutations (one line with a mutation in APP and two lines with different mutations in PSEN1) exhibited different phenotypes, particularly in Tau hyperphosphorylation, suggesting the capacity to model specific disease phenotypes from individual patients (Raja et al., 2016).

The need for improved *in vitro* models has been widely recognized for screening approaches, such as those used in drug development and toxicology (Frank et al., 2017; Bal-Price et al., 2018; Kim et al., 2019; Shafer et al., 2019). Drug development costs continue to rise, and it has long been reasoned that improved *in vitro* models of human physiology could lower these costs by improving preclinical studies and reducing failure rate of potential therapeutics in clinical trials (Begley and Ellis, 2012). Similarly, improved models would lead to increased detection sensitivity in toxicological screening assays, as these models would more accurately recapitulate physiology (Bal-Price et al., 2018; Kim et al., 2019). While microphysiological systems (e.g., engineered microfluidic devices, described in detail in the next section) have improved *in vitro* models and offer precise control over culture parameters, organoids provide macroscale architecture and organization that is difficult to recreate in traditional 3D culture systems (Bhatia and Ingber, 2014). As a tradeoff, organoid models sacrifice throughput—due to long culture times necessary for maturation—for this increased accuracy; however, researchers are implementing technological advances from more conventional systems, such as microfluidics and synthetic scaffolds, to increase throughput and efficacy of organoid models (Esch et al., 2015; Skardal et al., 2015, 2016).

## Technological Advances for Brain Organoid Development and Characterization

Advances in brain organoid complexity have come as a result of advances and new applications of various technologies. One such technology includes microfluidics, which allow for “organoids-on-a-chip” (Wang et al., 2018; Kim et al., 2019; Park et al., 2019). Microfluidics have been used to control the cellular microenvironment and engineer organ-on-a-chip systems, which recapitulate specific physiological aspects of particular organs and tissues (Bhatia and Ingber, 2014). While traditionally considered at-odds with organoids due to fundamental differences in engineering approaches (Jackson and Lu, 2016) and control (Bhatia and Ingber, 2014)—top-down approach and assembly of organ-on-a-chip models versus bottom-up approach and self-organization of organoids—researchers have recently begun combining the two approaches (Skardal et al., 2016; Takebe et al., 2017; Park et al., 2019). Microfluidic systems for brain organoid culture provide additional control of signaling molecules required for differentiation (i.e., morphogens) (Demers et al., 2016) and oxygen diffusion (Berger et al., 2018), which has long been recognized as a hurdle for brain organoid development (Lancaster et al., 2017). Improved control of morphogen gradients can be used to study developmental stages and differentiation at highly precise levels, such as motor neuron differentiation in the developing neural tube (Demers et al., 2016). By increasing oxygen diffusion throughout brain organoids via microfluidics, midbrain organoids exhibited reduced necrotic cores and increased numbers of dopaminergic neurons, highlighting increased differentiation efficiency (Berger et al., 2018). Ultimately, microfluidic devices provide precise control of many organoid parameters, such as size/shape (Ao et al., 2020) and media perfusion rate (including nutrient and growth factor supply) (Wang et al., 2018), leading to increased reproducibility (Yin et al., 2016; Wang et al., 2018; Kim et al., 2019; Park et al., 2019). Additionally, many microfluidic platforms are compatible with common imaging setups, allowing for live organoid imaging and monitoring (Yin et al., 2016; Kim et al., 2019). A microfabricated system was used to image and analyze folding dynamics of brain organoids over several weeks of development (Karzbrun et al., 2018a,b) and an organoid-on-a-chip model using controlled perfusion enabled assessment of developmental effects of nicotine exposure (Wang et al., 2018). These platforms have allowed for live imaging over time and throughout brain organoid development, as well as precise microenvironment control, leading to increased reproducibility when studying early development or developmental diseases and toxicity (Berger et al., 2018; Karzbrun et al., 2018a; Wang et al., 2018).

Biomaterials advances over the past decade have also contributed to development of improved organoids. Extracellular matrices and scaffolds are vital to stem cell self-renewal and differentiation, leading to the use of natural materials, such as Matrigel. However, Matrigel is not well-defined and can have considerable batch variation, prompting a need for defined scaffolds and materials (Yin et al., 2016). Defined biological materials have been widely used for neural tissue engineering (Boni et al., 2018; Kratochvil et al., 2019) and are beginning to show promise as scaffolds for brain organoid culture, as well. For example, brain organoids were generated in 10–14 days on composite hyaluronic acid-chitosan hydrogels in chemically defined media (Lindborg et al., 2016). These materials have many beneficial characteristics for brain organoid applications: they allow for simple and scalable organoid generation with high accessibility and applicability due to the lack of exogenous materials, they are widely available, they have a long history of neural biocompatibility, and they are amenable to growth factor loading and modification, if desired (Yang et al., 2015). In a similar strategy, hyaluronic acid-heparin hydrogels were shown to promote caudalization of brain organoids, demonstrating how various ECM components and factors can influence brain organoid development and function (Bejoy et al., 2018). In addition to biological materials, synthetic scaffolds and materials can be designed to mimic natural ECM mechanical properties and are tunable, providing precise control and mechanistic understanding of elements underlying neurogenesis and brain organoid development (Ranga et al., 2016). These scaffolds can also be loaded with various soluble factors to control signaling and the microenvironment, which contribute significantly to organoid development (Yin et al., 2016; Koo et al., 2019). Synthetic scaffolds that are chemically defined, scalable, and good manufacturing practices (GMP)-compliant—important for drug development and personalized medicine applications—have been specially designed to support 3D hPSC culture, allowing for expansion and simple passaging via thermoresponsive properties (Lei and Schaffer, 2013). Recently, similar synthetic, defined hydrogel scaffolds have been used to generate intestinal organoids comparable to those generated with Matrigel (Gjorevski et al., 2016; Cruz-Acuña et al., 2017; Gjorevski and Lutolf, 2017). These biomaterial advances—along with increased characterization of brain extracellular matrix and biomechanical properties—provide many capabilities to help design and engineer brain organoids.

In addition to bioengineering advances contributing to brain organoid development, considerable work has been done with genetic approaches to allow for precise genetic manipulation of organoids. Established genetic tools including adeno-associated viruses (AAVs), lentiviruses, electroporation, and CRISPR/Cas9 have been applied to brain organoids for a wide range of applications, from simple reporter expression to disease modeling (Fischer et al., 2019). This wide range of available tools can be utilized to obtain targeted spatiotemporal manipulation, for example, modifying all cells at an early stage or a specific subset of cells in a mature, developed organoid. These approaches have been used for various applications, from simple fluorescent labeling of neurons to study migration deficits in mutant organoids modeling lissencephaly (Bershteyn et al., 2017) to RNA knock-in or knockdown via electroporation to examine mechanisms of hypoplasia in microcephalic organoids (Lancaster et al., 2013; Li et al., 2017). In addition to these transient applications, CRISPR/Cas9 has been used to stably modify stem cell populations prior to organoid generation (Bershteyn et al., 2017; Li et al., 2017; Karzbrun et al., 2018a) or at specific time points, such as to introduce an oncogene to study glioblastoma in 4-months-old brain organoids (Ogawa et al., 2018). These examples demonstrate the considerable utility of genetic modifications for studying precise aspects of brain development and disease modeling, and how genetic approaches will continue to be vital to both organoid development and design, as well as characterization and mechanistic understanding. For an excellent recent review on genetic manipulation of brain organoids, see Fischer et al. (2019).

Along with genetic tools allowing researchers to characterize brain organoids and explore various mechanisms, -omics approaches have provided a much greater understanding of brain organoid development, both in healthy and disease states. Early organoids were characterized using common immunohistological markers, but in-depth characterization was limited (Lancaster et al., 2013; Lancaster and Knoblich, 2014). Recently, single cell RNA-sequencing (sc-RNA-seq) has unveiled the considerable diversity of cell types comprising brain organoids (Quadrato et al., 2017). The ability to analyze single cells across organoids, along with improved analytical methods [i.e., t-distributed stochastic neighbor embedding (tSNE)], has allowed researchers to examine cellular diversity at much higher detail (e.g., whole transcriptome compared to individual markers), at different developmental time points within organoids, and among organoids (Quadrato et al., 2017). Understanding this variability is important for improved organoid development and characterization, especially for disease modeling applications.

While promising, early organoid characterization left much to be desired in terms of depth—without understanding the extent of neuronal maturity and subtypes, cellular and regional interactions, and functional maturation, it is difficult to determine the usefulness of brain organoids as truly physiological models of disease (Quadrato et al., 2016). For example, a midbrain organoid with a high proportion of dopaminergic neurons could be useful for modeling Parkinson’s disease, but neuronal maturation and glia are also important components that may considerably affect degeneration and disease phenotype. To this end, sc-RNA-seq has begun to reveal the vast array of brain organoid cellular diversity and extent of maturation (e.g., dendritic spine formation) necessary for developing proper disease models (Quadrato et al., 2016, 2017).

Finally, a significant effort has been made to vascularize brain organoids in recent years. Without vascularization, significant cell death is observed in the inner regions of brain organoids, limiting proper development and analysis (Lancaster et al., 2013; Vargas-Valderrama et al., 2020). Several strategies have been employed to vascularize brain organoids. An initial strategy used implantation of brain organoids *in vivo*, resulting in host vascularization of the engrafted organoids, organoid maturation, and prolonged survival (Mansour et al., 2018). This approach has since been improved, incorporating endothelial cells to develop vascular structures *in vitro* prior to implantation, with implanted organoids developing more complex vasculature and integrating with host vessels, resulting in long-term survival and functional maturation (Pham et al., 2018; Shi et al., 2020). Notably, patient-derived hiPSCs were used to generate brain organoids and endothelial cells, supporting this approach to generate patient-specific vascularized brain organoids (Pham et al., 2018). Lastly, neural and endothelial co-differentiation has been observed in hESC-derived organoids, induced by vascular endothelial growth factor (VEGF) (Ham et al., 2020) or expression of an endothelial transcription factor, ETV2 (Cakir et al., 2019) early in brain organoid differentiation. Both approaches generated vascularized brain organoids exhibiting blood-brain barrier characteristics, and ETV2 expression increased neuronal activity and maturation (Cakir et al., 2019), suggesting significant value in disease modeling.

## Electrophysiological Analysis of Brain Organoids

The hallmark of functional analysis for neural cells and tissues, including brain organoids, is electrophysiology. The ability to record neuronal function is essential for many brain organoid applications, especially disease modeling and drug development (Sakaguchi et al., 2019). Most traditional electrophysiology techniques have been applied to brain organoids and have unique advantages and disadvantages (Poli et al., 2019).

Patch clamping allows researchers to record individual neurons in a brain organoid at high temporal resolution, providing detailed analysis of specific neurons (Pasca et al., 2015; Di Lullo and Kriegstein, 2017; Li et al., 2017; Cakir et al., 2019). The high temporal resolution is particularly useful for determining responses to specific perturbations, such as pharmacological treatment or optogenetic stimulation; however, as only individual neurons can be analyzed, little-to-no information on network connectivity or dynamics important to regional or global organoid function. To increase spatial resolution and analyze network activity, calcium imaging has been utilized (Lancaster et al., 2013; Sakaguchi et al., 2019). Calcium imaging overcomes these limitations of patch clamping, allowing for live cell imaging of neural activity in small groups of neurons. This is useful for analyzing specific regions of brain organoids and attempting to analyze synaptic activity and neural circuits (Sakaguchi et al., 2019). As a tradeoff, some of the high temporal resolution of patch clamping is lost. Additionally, the three-dimensionality of organoids poses challenges to acquiring calcium imaging data, as neurons must be oriented closely in the z-dimension to capture them in close succession and analyze connectivity patterns. While this may be acceptable for specific small regions, it limits global functional analysis.

Microelectrode arrays (MEAs) have been increasingly adopted for screening applications and other studies due to the ability to combine the temporal resolution of patch clamping with the network resolution of calcium imaging (McConnell et al., 2012; Cotterill et al., 2016; Frank et al., 2017; Shafer et al., 2019). By analyzing extracellular potentials from a relatively large array of electrodes simultaneously, many parameters of network connectivity can be assessed in real time. MEAs also offer significantly improved throughput compared to other recording techniques and many analytical tools have been developed for improved data analysis and interpretation (Egert et al., 2002; Pastore et al., 2016; Bridges et al., 2018). In organoids, this provides similar connectivity data as calcium imaging but on a much larger scale, allowing for entire region analysis or potential analysis of several organoid regions (Giandomenico et al., 2019). Recording across large portions of brain organoids has revealed strong connectivity between various regions within organoids, suggesting long-range neural circuits and inter-regional connectivity, instead of simply “nearest neighbor” connections (Giandomenico et al., 2019). As further evidence of these long-range circuits, brain organoids co-cultured with spinal cord explants were observed to project functional axon tracts toward the spinal cord explants that were able to stimulate muscle contraction (Giandomenico et al., 2019). As brain organoids become more complex and are used to model complex aspects of development and diseases, the ability to detect and analyze inter-regional connectivity and neural circuits across large distances becomes vital, and MEAs are useful tools to provide insight into these circuits. The large-scale recordings provided by MEAs are also amenable to combination with additional data sets or multiplexing with other assays. For example, correlation analysis of MEA activity throughout development with transcriptomics (sc-RNA-seq) and immunohistochemistry has provided mechanistic insight into developmental processes, such as simultaneous astrocyte population growth and neuronal maturation (Fair et al., 2020). Additionally, multiplexing MEA analysis with high content imaging can help offset concerns associated with brain organoid variability by increasing confidence in results reflected across modalities, supporting potential use for drug screening and other high-throughput approaches (Durens et al., 2020). Despite these advantages, however, MEAs are not without drawbacks. Most notably, the three-dimensionality of the organoids and planar electrode arrays typically limit recording to the outer edges of organoids, which may or may not be areas of significant interest. Recent advances to overcome this challenge are discussed in the next section.

Finally, optogenetics have been employed in conjunction with the above techniques to allow for precise stimulation and mechanistic studies (Shiri et al., 2019). Optogenetic manipulation has received widespread use in neuroscience and recent application in organoids to analyze and manipulate neural activity (Watanabe et al., 2017; Mansour et al., 2018). In particular, neuronal-specific channelrhodopsin expression in brain organoids was demonstrated (Watanabe et al., 2017), and optogenetic manipulation of implanted organoids in rodents was used to assess successful integration into the host brain, opening the door for vascularization strategies and disease modeling in a physiological microenvironment (Mansour et al., 2018). Despite relatively few applications in hiPSC-derived cells thus far, the potential for optogenetics to improve hiPSC and brain organoid models by allowing for deeper mechanistic analysis has been recognized (Chin and Goh, 2015; Su et al., 2015; Trujillo and Muotri, 2018). Relatively low transfection efficiency in hiPSC-derived cells compared to somatic cells may be partially responsible for the slow adoption of optogenetics in brain organoids and other hiPSC-derived cells; however, recent advances and comparisons of transfection techniques may help increase these studies moving forward (Chin and Goh, 2015; Rapti et al., 2015; Lee et al., 2019). Indeed, a neuromuscular junction (NMJ) model implementing hiPSC-derived neurospheres and muscle tissue was recently used to assess functional deficits in amyotrophic lateral sclerosis (ALS) (Osaki et al., 2018). This study suggests similar utility for functional disease modeling via optogenetics and brain organoids in the near future.

## Recent Advances in Electrophysiology—Applicability to Brain Organoids

While the electrophysiological techniques discussed above have been effective in providing functional data on brain organoids, the drawbacks of each method are notable, ultimately hindering the extent of functional analysis that can be performed. There have been many recent advances in electrophysiology that may provide improvements over these traditional methods, though these technologies are in their infancy or have yet to be applied to brain organoids. Some key advances with clear applicability for brain organoids are highlighted below and in Table 1.

**TABLE 1:** Electrophysiological advances with applicability to brain organoids.

Technology	Major advantage	Cellular resolution	Global resolution	Temporal resolution	Throughput*	Cultures used for validation	References
PatchSeq	Combines transcriptomics and electrophysiology at single-cell resolution	High (single cell)	High (cells can be analyzed from different regions of entire organoid)	High (20 kHz)	Low (single cell)	Primary mouse neocortical cells	Cadwell et al., 2016, 2017; Fuzik et al., 2016
						hESC/hiPSC-derived neurons	Chen et al., 2016
						Primary mouse hippocampal neurons	Földy et al., 2016
All-optical electrophysiology	Combines advantages of patch clamp and calcium imaging	High (single cell)	Medium (cells in a general region/cluster can be analyzed simultaneously, but constrained by imaging field)	Medium (1–2 ms)	Medium (clusters of cells within single imaging field)	Primary rat hippocampal neurons, hiPSC-derived neurons (iCell)	Hochbaum et al., 2014
						hiPSC-derived motor neurons (incl. SOD1-mutant)	Kiskinis et al., 2018
3DMEA	Allows three-dimensional recording (e.g., inner regions of organoids) as opposed to planar recording	Low (cannot correlate signals to individual cells, but can identify signals in 3D space)	High (3D probes allow for recording of large, identifiable regions throughout organoid)	High (10 kHz)	Very high (large number of cells throughout organoid with 256 recording channels)	3D hiPSC-derived neuron + astrocyte co-cultures	Soscia et al., 2020
Mesh nanoelectronics	Whole-organoid electrode coverage throughout all stages of development	Very low (low electrode density and ability to correlate signals to individual cells)	Very high (electrode coverage across entire organoid, but difficult to spatially control and locate specific electrodes)	High (10 kHz)	High (cells across organoid, but only 16 channels in current design)	Cardiac organoids	Li et al., 2019

*Throughput defined as how many cells can be recorded simultaneously, especially relative to other recording techniques.

A recently developed technique, dubbed PatchSeq, combines patch clamp electrophysiological recordings with sc-RNA-seq, allowing functional correlation to gene expression (Bardy et al., 2016; Cadwell et al., 2016, 2017; Chen et al., 2016; Földy et al., 2016; Fuzik et al., 2016; van den Hurk and Bardy, 2019). While still limited in scale and throughput, the correlation to genetic and morphological analysis provides a new dimension of functional analysis and could be extremely useful when analyzing particular subsets of neurons in brain organoids (Bardy et al., 2016; van den Hurk and Bardy, 2019). The authors found strong correlations between neuronal activity/maturation and 45 genes, some with known neuronal function, including synaptic plasticity and voltage-gated sodium channels. Interestingly, most of these genes had not previously been associated with neuronal function, representing potential new biomarkers for neuronal activity and maturation (Bardy et al., 2016). While this study was performed in mixed hiPSC-derived neuron and astrocyte co-cultures, this approach could reveal similar discoveries and associations about neurons in brain organoids, and perhaps more physiologically relevant biomarkers, as brain organoids contain more cell types and important three-dimensional organization.

Optogenetics has proven to be a useful tool for manipulation of neural activity, both in monolayer cultures and organoids. Traditionally, optogenetics has been utilized to stimulate and/or inhibit neurons of interest. Recently, the development of all-optical electrophysiology has provided a method to both manipulate and record neural activity at high spatiotemporal resolution (Hochbaum et al., 2014; Werley et al., 2017; Kiskinis et al., 2018). This method consists of co-transfecting neurons with both a channelrhodopsin (CheRiff) allowing for optogenetic stimulation and a spectrally orthogonal fluorescent genetically encoded voltage indicator (GEVI) (QuasAr) allowing for simultaneous recording of neural activity. Being able to stimulate and record simultaneously via this all-optical setup allows for network level recordings of neural circuits while maintaining both single-cell and high temporal resolution. Despite this promise, adoption began slowly due to relatively low construct expression levels and highly complex data analysis. More recently, however, improved analytical algorithms were developed to better extract activity and morphology data using this system and applied it to analyze human iPSC-derived motor neurons in a model of ALS (Kiskinis et al., 2018). There were clear differences between control and ALS cells, demonstrating its usefulness for both hiPSCs and disease modeling, which could be adapted to brain organoids. Particularly, ALS cells were hyperexcitable when unstimulated, as previously reported (Wainger et al., 2014), but the single-cell resolution afforded by optical electrophysiology showed hypoexcitability in response to strong stimulus. This highlights a key advantage of single-cell electrophysiology on larger populations of neurons, which would not be feasible with traditional patch clamping (Kiskinis et al., 2018). Ultimately, while it is still slightly less precise than patch clamping (1–2 ms vs. submillisecond temporal resolution), optical electrophysiology maintains much of the resolution afforded by patch clamping while significantly increasing throughput, which is important for the large-scale analysis needed for brain organoids.

**FIGURE 1:**
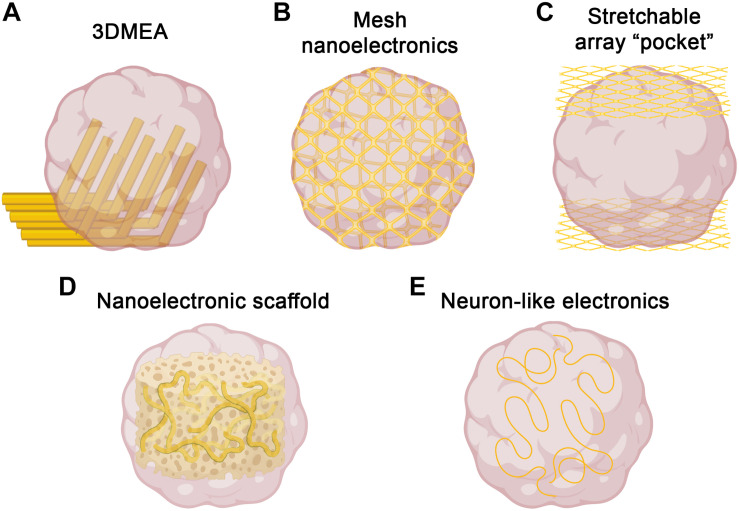
Strategies for three-dimensional electrophysiology. Several recent approaches to overcome challenges of electrophysiological recording in 3D could be used to record brain organoids. **(A)** 3DMEAs (Soscia et al., 2020) employ flexible, hinged probes to record inner regions of brain organoids. **(B)** Mesh nanoelectronics (Li et al., 2019) are integrated throughout organoids in early stages of development, allowing for whole-organoid longitudinal recording. **(C)** Stretchable polyimide “pocket” arrays (Shim et al., 2020) provide large-scale coverage of organoid surfaces, sacrificing inner region access for integration flexibility. **(D)** Nanoelectronics embedded into biomaterial scaffolds (Tian et al., 2012) offer tunability and biocompatible support for organoid growth, as well as integrated 3D recording. **(E)** Neuron-like electronics (Yang et al., 2019) mimic the size, shape, and mechanical properties of neurons, allowing for successful organoid incorporation, neural interfacing, and scaffolding to support neurogenesis/neuronal migration (figure created with Biorender.com).

The two-dimensionality of traditional MEAs is not an issue for typical monolayer cell culture; however, the three-dimensionality of brain organoids significantly limits accessibility to the majority of cells in the organoid. MEAs have still been useful to date, but recordings must be performed in a single plane, usually at the edge of the organoid. To overcome this, three-dimensional MEAs (3DMEAs) are currently being developed (Soscia et al., 2020; Figure 1A). By incorporating electrodes into flexible, hinged probes, extracellular recordings can be taken from 3D neural networks, such as those found in organoids. Importantly, these devices are compatible with many existing readily accessible recording setups, thus facilitating rapid adoption by brain organoid researchers. 3D hiPSC-derived neural cultures were recorded over 38 days *in vitro* (DIV), observing similar activity as other recording methods, suggesting viability and long-term biocompatibility without sacrificing recording ability or resolution. The culture analyzed was a mixed neuron-astrocyte co-culture suspended in hydrogel, which—while less complex than organoids—demonstrates the ability to record from similar 3D cultures and neural networks, as well as to spatially map neural activity in three dimensions. A high-density 3DMEA platform may allow for more precise spatial mapping of active neurons (Yuan et al., 2020). Indeed, another high density MEA platform was recently used to assess activity in retinal organoids, demonstrating applicability to organoid cultures (Georgiou et al., 2020). This same MEA platform was also used in a similar manner to compare differentiation protocols for retinal organoid development (Mellough et al., 2019), further demonstrating utility for quick, simple quantification of organoid activity. Traditional MEA systems provide network-level information at the cost of single cell-resolution due to relatively low electrode density, making it difficult, if not impossible, to correlate specific signals with individual neurons. With increased electrode density, single-cell activity analysis can be performed, providing both network dynamics and changes in individual cells, allowing for neural circuit mapping and analysis of how circuit connectivity changes over time (Yuan et al., 2020). This detailed connectivity analysis in brain organoids could reveal developmental insights (i.e., regional interconnectivity) or insight into neurodegeneration or synaptic rearrangement typical of diseases such as ALS or Alzheimer’s disease. These applications to organoids should be feasible, providing an avenue to record the inner regions of brain organoids, which has to date been elusive.

An alternative approach to obtain three-dimensional recordings of brain organoids is the use of electrodes embedded into a stretchable mesh. These “mesh nanoelectronics” can be integrated with cell monolayers in the early stages of organoid development, after which they have the ability to stretch as the organoids develop into three-dimensional structures, essentially taking the shape of the entire organoid (Li et al., 2019; Figure 1B). The unique advantage of this approach is that by the time the organoid finishes developing, it consists of evenly spaced electrodes across the entire structure. Additionally, this provides the ability to record from the organoid across all stages of development, assessing neural ontogeny and the onset of activity. The researchers demonstrated this ability, as well as long-term biocompatibility, via integration into cardiac organoids. By recording throughout organogenesis, researchers were able to determine organoid maturation state by measuring synchronized bursting patterns, which would be difficult or impossible to measure with traditional recording techniques. Combined with no observable changes in marker expression throughout development, this suggests device implantation does not interfere with typical developmental processes, including sarcomere assembly. A similar approach was recently reported, in which stretchable polyimide arrays were sandwiched around brain organoids, creating a stretched “pocket” capable of conforming to the organoids (Shim et al., 2020; Figure 1C). This approach is more limited to the outside surface of the organoid, but may provide more flexibility with timing and application, as it does not need to be integrated from the beginning of organoid development. As with the 3DMEAs, electrode density can be improved, but the potential for whole-organoid recordings, especially throughout development, represents a significant improvement over traditional electrophysiological methods.

While the above approaches address challenges associated with recording 3D brain organoids, considerable improvements are also being made regarding materials and electrode designs to enhance both recording capabilities and biocompatibility (Didier et al., 2020). Recent advances in bioprinting have been utilized to demonstrate proof-of-concept for patterning and printing MEAs on soft material substrates with mechanical properties similar to brain tissue, assisting with biocompatibility (Adly et al., 2018; Borda et al., 2020). To improve electrode properties (i.e., biocompatibility, impedance, structural integrity, transparency), many new materials have been used for design and coating, such as indium tin oxide, gold, titanium nitride, and ruthenium oxide, among others (Jahnke et al., 2019; Koklu et al., 2019; Ryynänen et al., 2019, 2020; Atmaramani et al., 2020). The continued development of MEAs incorporating these and other materials, as well as improved incorporation methods, may help improve signal-to-noise ratios and fidelity for brain organoid recordings.

Bioinspired electrodes and scaffolds also have significant potential to improve recording capabilities in brain organoids (Li et al., 2020). By integrating nanoelectronics into biomaterials, nanoelectronic scaffolds (nanoES) were created and used to support 3D neural cultures (Tian et al., 2012; Figure 1D). These nanoES have macroporous structures, mimicking natural extracellular matrices and allowing for unimpeded neurite outgrowth and integration, as well as good biocompatibility. By modifying this approach with various biomaterials and synthetic scaffolds, nanoES are tunable, increasing the potential for incorporation into brain organoids. Another bioinspired approach was the recent design of neuron-like electronics (NeuE)—neural probes that mimic the size, shape, and mechanical properties of neurons for high resolution and integration into neural tissues (Yang et al., 2019; Figure 1E). The mechanical properties of these neurite-diameter probes allow for significantly reduced stiffness compared to most other flexible electrodes, supporting neuronal interfacing. These interfaces are stable over time, allowing for chronic recording, and easily able to be multiplexed with 3D imaging. Notably, NeuE implanted into mouse brains also enhanced endogenous neural progenitor cell migration, providing similar scaffolding properties as radial glia cells, suggesting the potential to enhance neural development in brain organoids while also providing high resolution electrophysiological recording.

Finally, in addition to the physical limitations of recording brain organoids in three dimensions, there are also challenges associated with data processing and analysis. Traditional electrophysiological data processing is typically carried out in a similar fashion, regardless of recording method. For action potential (spike) and burst analysis, recorded signals are high pass filtered, followed by spike detection and sorting, and finally parameter calculation from the analyzed spike patterns (Robinette et al., 2011; Latchoumane et al., 2018). Many labs perform these steps with custom scripts, but there is also a litany of commercial and open source software solutions available (Egert et al., 2002; Pastore et al., 2016; Bridges et al., 2018; Unakafova and Gail, 2019), which may be useful when adapting these analyses to 3D recordings, especially for researchers without a strong background in electrophysiology that may be analyzing brain organoids. Spike detection is typically threshold-based to detect spikes over baseline noise, and spike sorting often consists of cluster analysis to separate spikes based on waveform shape corresponding to individual neurons detected by the same electrode (Hilgen et al., 2017). This is a crucial technique for three-dimensional analysis, especially when high spatial resolution and individual neuronal signal isolation is necessary (e.g., connectivity mapping, spike timing analysis) (Regalia et al., 2016; Hilgen et al., 2017; Yger et al., 2018). Spike sorting can be computationally intensive for 2D analysis and may be much more difficult in 3D, as increased channels and spiking events increases computation exponentially (Hilgen et al., 2017). Additionally, with more electrodes in the vicinity of each other due to the third dimension, more electrodes must be considered when attempting to distinguish spikes across multiple electrodes (Yger et al., 2018). To address these issues, solutions have been developed implementing improved spike detection, dimensionality reduction, and pre-defined templates to achieve lower error rates—and thus, reduced necessity for manual oversight which becomes impossible with high-density arrays (Lefebvre et al., 2016; Hilgen et al., 2017)—as well as template-matching, where spikes are assumed to adhere to pre-defined “template” waveforms, reducing computational cost (Marre et al., 2012). Another promising approach involves utilizing probe geometry and knowledge of the spatial relationship between electrodes to optimize analysis (Rossant et al., 2016), which would be useful in 3D arrays with relatively determinable electrode locations within a brain organoid (e.g., the 3DMEAs highlighted above).

## Outlook

Brain organoids present enormous potential for disease modeling and understanding the human brain and represent a culmination of many advancements in stem cell biology. To improve the applicability of brain organoids, knowledge can also be taken from advances realized in other organoid models. For example, improved characterization approaches have been applied to other organoid models, such as single molecule fluorescent in situ hybridization (smFISH). This technique provides single-cell analysis while preserving spatial information, as it is performed in situ without disrupting the three-dimensional architecture of the organoids (Omerzu et al., 2019). While fewer transcripts can be analyzed at once, the spatial information can provide insight and context into the activity of individual cells in specific organoid regions. This has recently been applied to colon organoids to assess Wnt signaling pathway alterations across entire organoids and regional transcription differences between crypt structures and the main organoid body (Omerzu et al., 2019); this could prove highly useful for analyzing cell signaling throughout developmental stages in brain organoids, especially development and integration of multiple regions. smFISH was also used to analyze post-transcriptional regulation in whole Drosophila brains, demonstrating its utility for detailed mechanistic analysis not feasible with immunohistochemistry (Yang et al., 2017).

Despite the significant advances and rapid evolution in brain organoid generation and engineering in recent years, reliable functional output and analysis is vital for brain organoid applications, especially disease modeling, and has lagged behind other organoid characterization, such as immunohistochemistry and transcriptomics (Wang, 2018; Poli et al., 2019). Just as *in vivo* models typically require overt behavioral phenotypes relevant to the diseases they are modeling, brain organoids cannot truly be considered representative of the human brain without physiologically relevant function—in this case, neural activity.

Electrophysiological advances for *in vivo* recording, such as Neuropixels (Jun et al., 2017; Steinmetz et al., 2018), and human medicine (e.g., neuroimaging) (Lau-Zhu et al., 2019) are opening the door to analyze highly specific populations of neurons and brain regions at high spatiotemporal resolution. For example, Neuropixels probes have the capability to record entire pathways consisting of several brain regions *in vivo* simultaneously, such as the primary motor cortex and striatum (Steinmetz et al., 2018). The 3DMEAs highlighted in this review present similar opportunities for *in vitro* analysis of brain organoids, such as the potential to record, say, dopaminergic neurons in both a substantia nigra-like region and ventral tegmental area-like region of a midbrain organoid to study Parkinson’s disease. These areas are differentially effected in Parkinson’s disease (Smits and Schwamborn, 2020), representing a clear application for brain organoid modeling.

Advances over the past several decades in stem cell biology, synthetic biology, and bioengineering have coalesced into a large umbrella of multi-cellular engineered living systems (M-CELS) (Kamm et al., 2018). M-CELS, including organoids, microphysiological systems, and “biobots”—machines consisting of biological material as building blocks and actuators—is a quickly developing field that could benefit significantly from advances in brain organoid analysis. One example of a potential M-CELS therapeutic is a biological pump, consisting of an endothelial vessel surrounded by muscle, which is innervated and controlled by a brain organoid (Kamm et al., 2018). This pump could be used for *in vitro* models of cardiovascular function or potentially implanted for regenerative medicine applications. While M-CELS as a whole is still in its infancy, rapid advances in underlying fields and technologies (including many of those described here) are enabling the realization of many of these systems. Improvements in brain organoid analysis and control, such as the electrophysiological advances described in this review, could help design M-CELS with the ability to sense and process their surroundings, leading to autonomous function via neural logic and computation.

Improved recording also introduces analytical considerations. While each of these methods presents promise for brain organoid analysis, each method also presents new analytical challenges. All-optical electrophysiology requires complex image analysis, electrophysiological parameter extraction, and statistical analysis, hindering early adoption (Kiskinis et al., 2018). 3DMEAs will likely require similar changes to existing analytical pipelines to account for the increased spatial information during processing (e.g., spike sorting). “Big data” has become a common challenge in neuroscience as technologies improve (including the ones mentioned above, sc-RNA-seq, and others), large multi-institutional projects are launched (e.g., the Brain Research through Advancing Innovative Neurotechnologies (BRAIN) Initiative), and researchers have access to high-performance computing (Chen et al., 2019; Qu et al., 2019). These advances, combined with continued dimensionality reduction, deep learning, and other “big data” approaches to traditional methods will help facilitate adaptability and utility of these methods.

**FIGURE 2:**
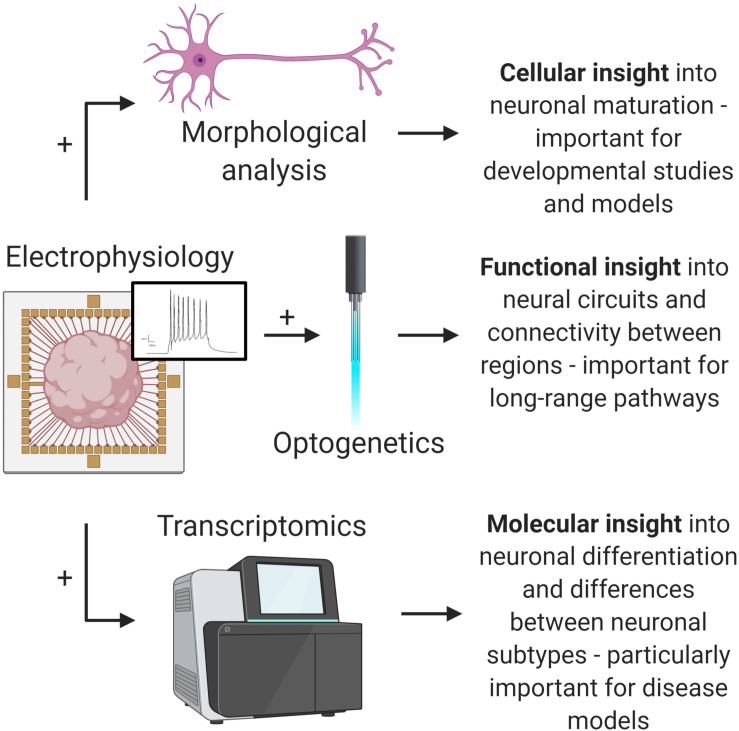
Multi-omic analysis of brain organoids. Combining advanced electrophysiological methods with existing methods will provide valuable insights into important brain organoid applications. Electrophysiology and morphological analysis (top) allows for increased understanding of neural maturation and development. Electrophysiology and optogenetics (middle) allows for complex pathway analysis. Electrophysiology and transcriptomics provides insight into detailed functional phenotypes and differences between neuronal subtypes, which is important for disease modeling (e.g., dopaminergic neurons in Parkinson’s disease models). Ultimately, advances in electrophysiology will open up these and other analytical possibilities (figure created with Biorender.com).

Ultimately, the approaches highlighted here represent a new wave of brain organoid functional analysis. By incorporating these with existing organoid technologies, such as optogenetics and sc-RNA-seq, researchers can extract more valuable information from brain organoids (Figure 2). The rapid development and evolution of these technologies continues to move closer to the goal of comprehensive organoid characterization—correlation of both identity and function of individual cells comprising brain organoids. These approaches will hopefully help unlock the full potential of brain organoids for developmental studies, disease modeling, and drug discovery applications.

## Author Contributions

AP and SS designed, wrote, and edited the manuscript and have approved it for publication. Both authors contributed to the article and approved the submitted version.

## Conflict of Interest

The authors declare that the research was conducted in the absence of any commercial or financial relationships that could be construed as a potential conflict of interest.
